# Triggering receptor expressed on myeloid cells-1 expression on monocytes is associated with inflammation but not with infection in acute pancreatitis

**DOI:** 10.1186/cc7876

**Published:** 2009-05-14

**Authors:** Eduardo Ferat-Osorio, Isabel Wong-Baeza, Noemí Esquivel-Callejas, Silvia Figueroa-Figueroa, Andrés Duarte-Rojo, Gilberto Guzmán-Valdivia-Gómez, Heriberto Rodea-Rosas, Rubén Torres-González, Patricio Sánchez-Fernández, Lourdes Arriaga-Pizano, Constantino López-Macías, Guillermo Robles-Díaz, Armando Isibasi

**Affiliations:** 1 Medical Research Unit on Immunochemistry, Specialties Hospital. National Medical Centre "Siglo XXI". Mexican Institute for Social Security (IMSS), Mexico City, Mexico; 2 Gastrointestinal Surgery Department, Specialties Hospital. National Medical Centre "Siglo XXI". Mexican Institute for Social Security (IMSS), Mexico City, Mexico; 3 PhD Program on Biomedical Sciences, Facultad de Medicina, Universidad Nacional Autónoma de México (UNAM), Mexico City, Mexico; 4 Immunology Department, National School of Biological Sciences, National Polytechnic Institute, Mexico City, Mexico; 5 General Surgery Department, General Hospital of Mexico, Mexico City, Mexico; 6 Pancreatic Unit, National Institute of Medical Sciences and Nutrition "Salvador Zubirán", Mexico City, Mexico; 7 General Surgery Department, Regional General Hospital "Carlos MacGregor Sánchez Navarro", IMSS, Mexico City, Mexico; 8 Medical Unit of High Specialization (UMAE), "Dr. Victorio de la Fuente Narváez", IMSS, Mexico City, Mexico; 9 Liver, Pancreas and Motility Laboratory (HIPAM), Experimental Medicine Department, Facultad de Medicina, Universidad Nacional Autónoma de México, Mexico City, Mexico

## Abstract

**Introduction:**

Acute pancreatitis (AP) is usually a mild and self-limiting disease, but some patients develop a severe form that is associated with high mortality. In AP, local inflammation is followed first by the systemic inflammatory response syndrome and then by the compensatory anti-inflammatory response syndrome, which is defined by low human leukocyte antigen (HLA)-DR expression on monocytes, increased concentration of the anti-inflammatory cytokine IL-10, and decreased monocyte function. Our aim was to measure the expression of triggering receptor expressed on myeloid cells (TREM)-1 (a proposed marker of infection or inflammation) and HLA-DR on monocytes, and the serum concentrations of IL-6 (a proinflammatory cytokine) and IL-10 in patients with AP to determine whether these markers can identify patients at high risk of developing severe AP or infection.

**Methods:**

Fifty healthy volunteers, 18 patients with mild AP, and 11 patients with severe AP were included in this study. Samples were taken at admission and one and three days later. TREM-1 and HLA-DR expression was evaluated by flow cytometry, and soluble TREM-1, IL-6 and IL-10 concentrations were measured by ELISA.

**Results:**

TREM-1 expression was higher in patients with AP than in healthy volunteers, but there was no difference between patients with mild and severe AP. TREM-1 expression was not associated with mortality or with the presence of infection. Soluble TREM-1 concentration in serum was higher in non-survivors than in survivors. HLA-DR expression was lower and IL-6 concentration higher in patients with severe AP and in infected patients.

**Conclusions:**

Increased TREM-1 expression was associated with the presence of inflammation but not infection in AP. In patients with AP, low HLA-DR expression and high IL-6 concentration could predict severity and infection in samples taken shortly after admission.

## Introduction

Inflammation is essential for survival, but it can also be an important cause of morbidity and mortality. One example of the deleterious effects of inflammation is acute pancreatitis (AP). Although AP is usually a mild and self-limiting disease, 20% to 31% of affected patients develop severe disease, and mortality rates can reach 25% in cases of infected pancreatic necrosis [1,2]. Intrapancreatic activation of digestive enzymes causes local tissue damage and the release of proinflammatory mediators by resident macrophages and acinar cells [3]. Proinflammatory cytokines are produced initially in the pancreas, and later in the liver, lungs, and spleen. The mechanism causing this secondary cytokine production is unknown [4]. The systemic release of proinflammatory mediators in AP causes a generalized inflammatory response in sites remote from the initial injury site and gives rise to the systemic inflammatory response syndrome (SIRS) [5,6].

Patients who progress to severe AP have a high mortality rate during their first week of evolution due to multiple organ failure. Those who survive frequently develop extensive necrosis of pancreatic and peripancreatic tissues [7], and 30% to 70% of the latter become infected. In these infected patients, multiple organ failure and death can ensue [8,9].

Infecting microorganisms contain pathogen-associated molecular patterns (PAMPs) that are recognized by the innate immune system and increase the production of adhesion molecules, proinflammatory cytokines, acute-phase proteins, nitric oxide synthase, cyclooxygenase-2, and triggering receptors expressed on myeloid cells-1 (TREM-1), among others [10]. TREM-1 is expressed on neutrophils and monocytes, and signaling through TREM-1 induces the secretion of proinflammatory cytokines and chemokines, and the expression of costimulatory molecules [11]. This secondary inflammatory response, or 'second-hit' response, can lead to tissue damage and can orchestrate organ failure after the first week of AP [12].

To restore homeostasis, the proinflammatory response is compensated by anti-inflammatory mediators such as IL-10 and soluble receptors that suppress the synthesis or the effects of proinflammatory cytokines [13]. During SIRS, an anti-inflammatory response can develop, leading to what Bone [6] defined as the compensatory anti-inflammatory response syndrome (CARS). SIRS is defined by clinical parameters [5], while CARS is defined on molecular grounds by low levels of major histocompatibility class II human leukocyte antigen (HLA)-DR molecules on blood monocytes and by low production of TNF-α when monocytes are challenged with PAMPs *ex vivo *[14].

TREM-1 was initially proposed as an early marker of infection because its expression is high in peritoneal neutrophils of septic shock patients [11]; a soluble form of TREM-1 is present in high concentrations in bronchoalveolar lavage of patients with pneumonia [15]; and soluble TREM-1 concentration is high in the serum of septic patients [16]. However, other studies have reported that TREM-1 expression increases in noninfectious pathologies [17,18], and our previous results have shown that the expression of this molecule increases after surgery, particularly in patients with preexisting SIRS, but without infection [19]. In patients with AP, high levels of TREM-1 mRNA correlate with increased severity of the disease [20]. Cytokine analysis in patients with AP has attempted to establish markers of severity (IL-6 or IL-8) or markers of progression (TNF-α or IL-1β) [21,22].

The aim of our present study was to measure the levels of TREM-1 and HLA-DR on monocytes, and the serum concentrations of IL-6 and IL-10 in patients with AP, and to determine whether these markers can be used for early identification of patients at high risk of developing severe AP or infection.

## Materials and methods

### Patients and controls

Twenty-nine patients from four hospitals (two general and two referral hospitals) were included in this study, which was approved on 13 September, 2006, by the Ethics and Research Committee from each hospital and by the National Committee for Scientific Research (No. 2006-785-080). The patients or their legal representatives received detailed information about this protocol, and, if they chose to participate, signed an informed consent form.

All patients between 18 and 80 years with confirmed AP were suitable to enter the study. AP diagnosis was based on the presence of typical clinical symptoms and at least a threefold increase in serum amylase or lipase concentration, and was classified as mild or severe according to the Atlanta Criteria [2,23]. Patients with more than 72 hours of evolution or patients who had an exploratory laparotomy performed within this time of evolution were not included. Other noninclusion criteria were pregnancy; treatment with immune suppressors or chemotherapy; HIV, hepatitis B virus or hepatitis C virus infection; or the presence of neoplastic or autoimmune diseases. A group of 50 healthy volunteers (blood bank donors) was also included in this study for comparison purposes.

### Blood samples

In patients with AP, blood samples were drawn within 24 hours of admission (day 0), and one and three days later. One sample was collected in an anticoagulant-free tube and another in a lithium heparin-containing tube (4 ml each). In healthy volunteers, the same samples were obtained on a single occasion. Anticoagulant-free blood samples were centrifuged at 2500 rpm for 10 minutes, and the serum was removed, and stored in aliquotes at -70°C until cytokine quantification. The lithium heparin blood samples were processed immediately for flow cytometry.

### Antibodies

Fluorescein isothiocyanate (FITC)-labeled anti-CD14 monoclonal antibody and phycoerythrin (PE)-cyanine dye Cy5-labeled anti-HLA-DR monoclonal antibody were purchased from BD Biosciences Pharmingen (San Jose, CA, USA; clone L243, mouse IgG2a, κ; and clone M5E2, mouse IgG_2a_, κ; respectively). PE-labeled anti-TREM-1 monoclonal antibody was obtained from R&D Systems (Minneapolis, MN, USA; clone 193015, mouse IgG1). This combination of fluorochromes allowed us to perform triple staining on each sample to measure TREM-1 and HLA-DR expression on CD14^high ^cells. In monocytes from healthy volunteers, 83.31% ± 11.27% of these cells expressed MHC-II, and the mean fluorescence intensity (MFI) of TREM-1 was 343.5 ± 155. PE-labeled mouse IgG1 and FITC-labeled mouse IgG2a were used as isotype-matched controls.

### Flow cytometry

In a polystyrene tube (BD Biosciences, San Jose, CA, USA), 50 μl of heparinized whole blood was mixed with 3 μl each of anti-CD14, anti-HLA-DR, and anti-TREM-1 antibodies, or the appropriate isotype controls, and incubated for 20 minutes at 4°C. Then, 250 μl of BD FACS lysing solution 1× (BD Biosciences, San Jose, CA, USA) was added, the cells were incubated for 10 minutes, and the stained cells were washed, resuspended, and analyzed for three-color immunofluorescence by flow cytometry (FACS Aria, Becton Dickinson, Franklin Lakes, NJ, USA). Cells with CD14^high ^expression were selected from a side scatter vs CD14/FITC dot plot. From this gated region, cells expressing TREM-1/PE or HLA-DR/PerCPCy5 were selected, using isotype controls as reference, and MFI or percentage values of the selected cells were taken. At least 5000 events in the CD14^high ^region were analyzed. Data analysis was performed using FACS Diva software version 4.1 (Becton Dickinson, Franklin Lakes, NJ, USA).

### Soluble TREM-1 and cytokine quantification

The concentration of soluble TREM-1 was measured in previously aliquoted serum samples using an ELISA kit (R&D Systems Minneapolis, MN, USA), according to the manufacturer's protocol. The concentrations of IL-6 and IL-10 were measured in previously aliquoted serum samples using ELISA kits (BD Biosciences Pharmingen, San Jose, CA, USA), according to the manufacturers' protocols.

### Statistical analysis

Data are represented on box and whiskers graphs, which depict median and 5% to 95% percentiles. Data were analyzed by Kruskal-Wallis test with Dunn's *post hoc *test using GraphPad Prism version 5.0 (GraphPad Software, Inc., La Jolla, CA, USA). Statistical significance was set at *P *< 0.05.

## Results

### Demographic data of patients and controls

Twenty-nine patients with AP were included in this study. Their average age was 43 years (range, 17 to 79 years); 18 were women and 11 were men. Twenty-two patients had AP of biliary origin (75%), one patient had AP caused by alcohol consumption (4%), one patient had AP caused by hypertriglyceridemia (4%), and five patients had idiopathic AP (17%). Eighteen patients had mild AP (62%), and 11 patients had severe AP (38%). One patient with mild AP was discharged from the hospital due to complete resolution of AP before the third blood sample was taken.

Five patients with severe AP died; one patient developed respiratory insufficiency and died two days after admission to the hospital; this patient died before the second blood sample had been collected. The other four patients with severe AP that died developed infections. One patient had pancreatic abscess caused by *Enterococcus faecalis *(diagnosed on day 23, deceased on day 41). Another patient with AP from biliary origin developed acute cholecystitis complicated with emphysematous cholecystitis 2 days after the onset of AP, and during the emergency laparotomy, purulent material was found in abdominal cavity and pancreas inflammation was confirmed (deceased on day 7). Two patients developed pneumonia; one patient with *Acinetobacter baumannii *and *Escherichia coli *in bronchoalveolar lavages (diagnosed on day 7, deceased on day 20), and the other with clinical and radiological diagnosis (diagnosed on day 4, deceased on day 5). A fifth patient with severe AP developed urinary infection with *Pseudomonas aeruginosa *(diagnosed on day 18). So, one severe AP patient developed infection during the period in which the blood samples were being collected (days 0 to 3), and four patients with severe AP developed infection after this period.

Fifty healthy volunteers (blood bank donors) were also included in this study; their average age was 34 years (range, 19 to 53 years); 11 were women and 39 were men.

### TREM-1 expression is higher in patients with AP, but this increase is not associated with mortality or with the presence of infection

**Figure 1:**
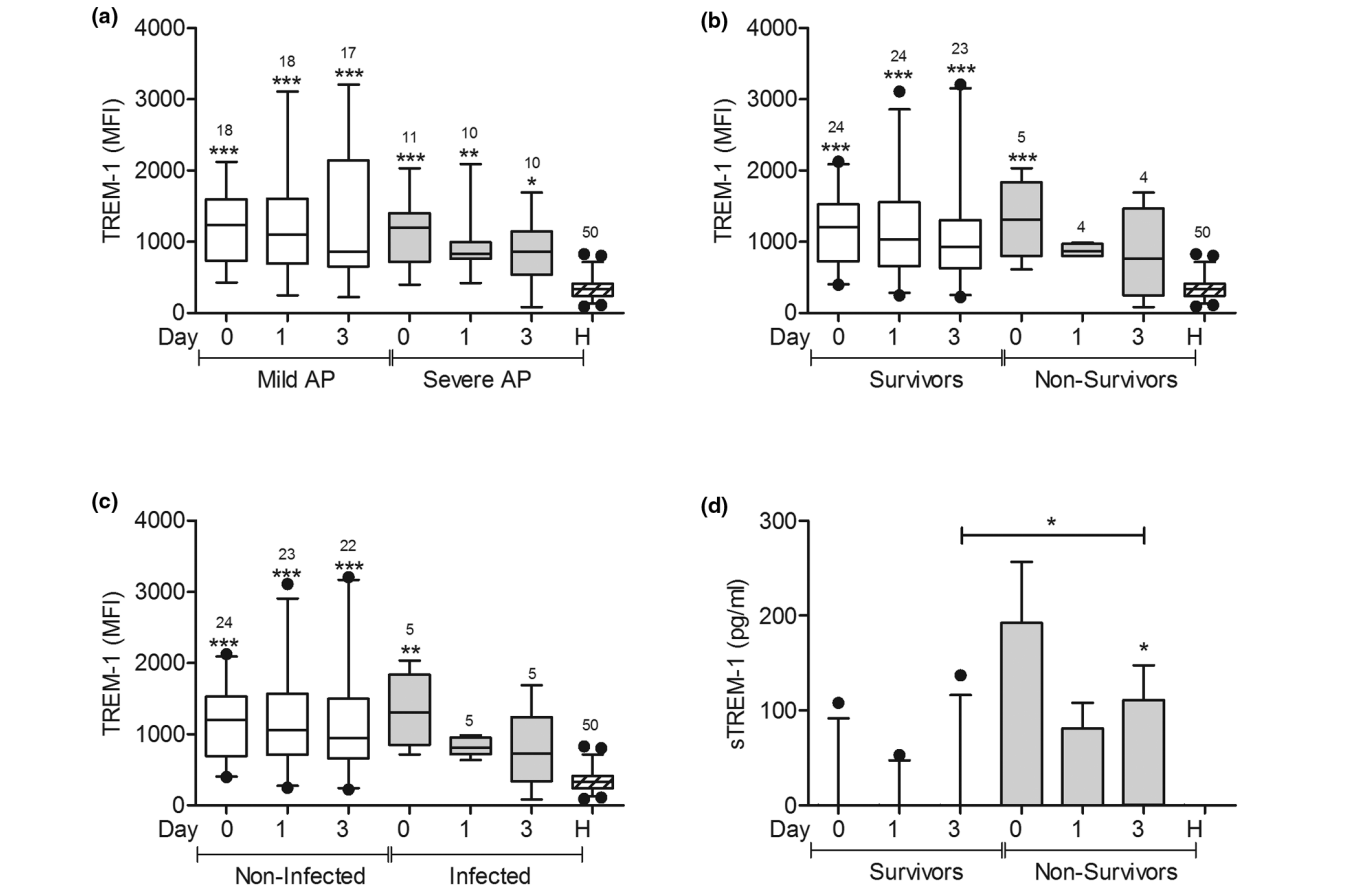
TREM-1 expression was higher in patients with AP, but this increase was not associated with mortality, or with the presence of infection.  **(a) **Triggering receptor expressed on myeloid cells-1 (TREM-1) expression was measured on blood monocytes from patients with mild acute pancreatitis (AP; n = 18), patients with severe AP (n = 11), and healthy volunteers (n = 50). Samples were taken from patients on admission (day 0) and one and three days later. One patient with mild AP was discharged from the hospital before the third blood sample was taken, and one patient with severe AP died after the first blood sample was collected. **(b) **Patients with AP were grouped according to survival (24 of these patients survived and 5 died). **(c) **Patients with AP were grouped according to the presence of infection (5 of the 29 patients developed infection). **(d) **Soluble TREM-1 expression was measured in the serum of patients with AP, which were grouped according to survival. * *P *< 0.05, ** *P *< 0.01, *** *P *< 0.001; the signs over each bar represent comparisons vs. healthy volunteers (H). MFI = mean fluorescence intensity.

TREM-1 expression was significantly higher in all patients than in healthy volunteers at each of the three times (Figure 1a). However, the expression levels of TREM-1 did not differ between patients with mild and severe AP (Figure 1a), between survivors and non-survivors (Figure 1b), or between infected and non-infected patients (Figure 1c). Non-survivors had higher soluble TREM-1 concentrations in serum than survivors on day 3 (Figure 1d). The concentrations of soluble TREM-1 did not differ between patients with mild and severe AP or between infected and non-infected patients with AP (not shown).

### HLA-DR expression is lower in patients with severe AP and in infected patients

**Figure 2:**
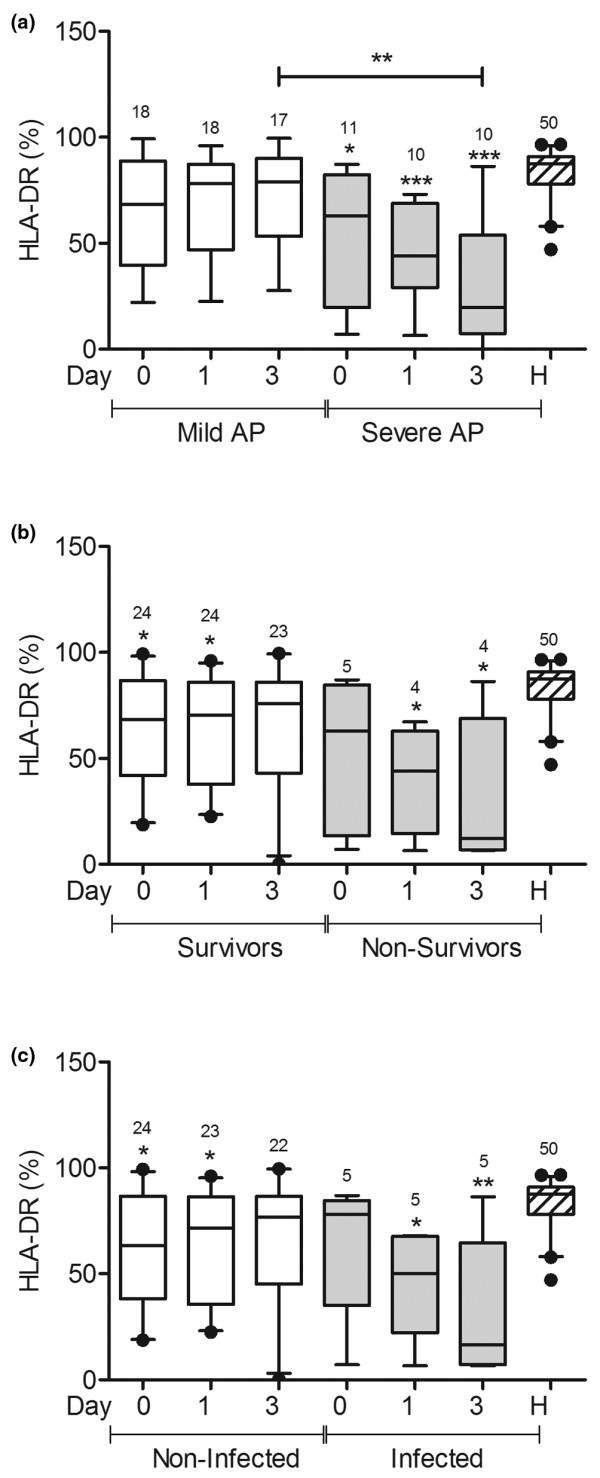
HLA-DR expression was lower in patients with severe AP and in infected patients **(a) **Human leukocyte antigen (HLA)-DR expression was measured on blood monocytes from patients with mild acute pancreatitis (AP; n = 18), patients with severe AP (n = 11), and healthy volunteers (n = 50). Samples were taken from patients on admission (day 0) and one and three days later. One patient with mild AP was discharged from the hospital before the third blood sample was taken, and one patient with severe AP died after the first blood sample was collected. **(b) **Patients with AP were grouped according to survival (24 of these patients survived and 5 died). **(c) **Patients with AP were grouped according to the presence of infection (five of the 29 patients developed infection). * *P *< 0.05, ** *P *< 0.01, *** *P *< 0.001; the signs over each bar represent comparisons vs. healthy volunteers (H).

HLA-DR expression was lower in patients with severe AP than in healthy volunteers at all times, and HLA-DR expression was lower in patients with severe AP than in patients with mild AP three days after admission (Figure 2a). The expression of HLA-DR was lower in non-survivors than in healthy volunteers on days 1 and 3 (Figure 2b). The expression of HLA-DR was also significantly lower in infected patients three days after admission than in healthy volunteers (Figure 2c).

### IL-6 and IL-10 concentrations were higher in patients with severe AP and in infected patients

**Figure 3:**
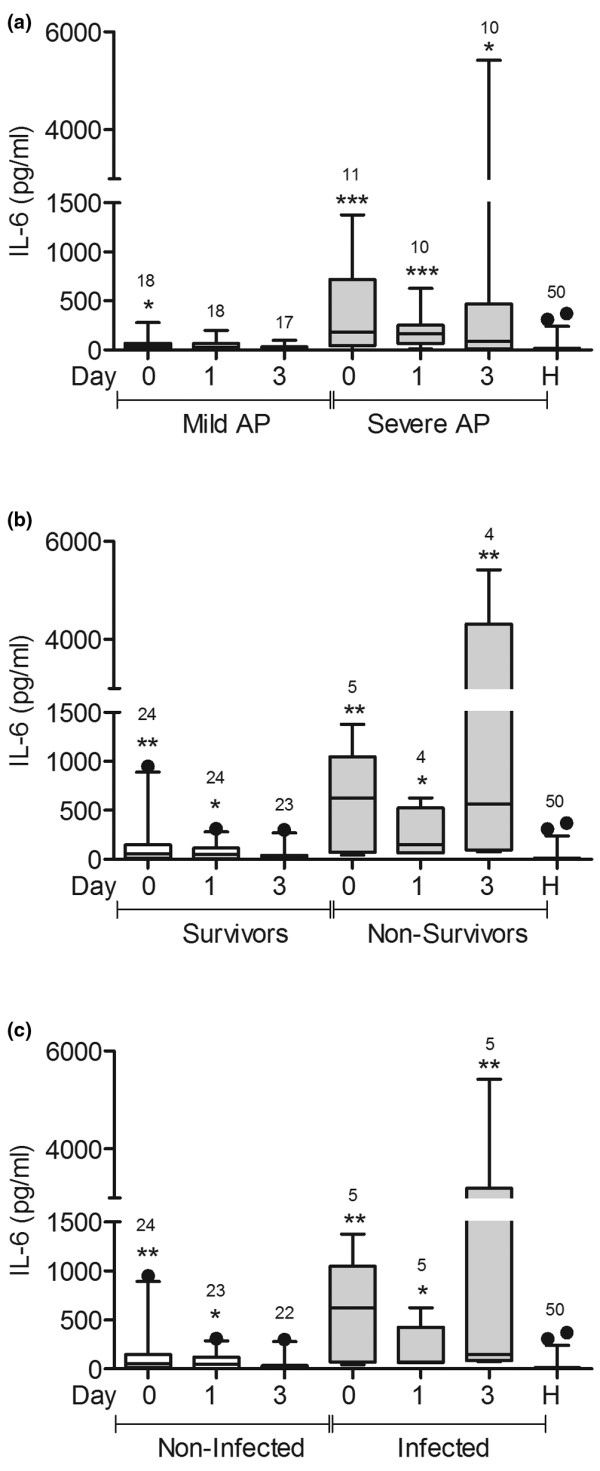
IL-6 concentration was higher in patients with severe AP and in infected patients. IL-6 concentration was measured in serum from patients with mild acute pancreatitis (AP; n = 18), patients with severe AP (n = 11), and healthy volunteers (n = 36). Samples were taken from patients on admission (day 0) and one and three days later. **(a) **Patients with mild and severe AP, **(a) **survivors and non-survivors, and **(c) **infected and non-infected patients are shown. * *P *< 0.05, ** *P *< 0.01, *** *P *< 0.001; the signs over each bar represent comparisons vs. healthy volunteers (H).

**Figure 4:**
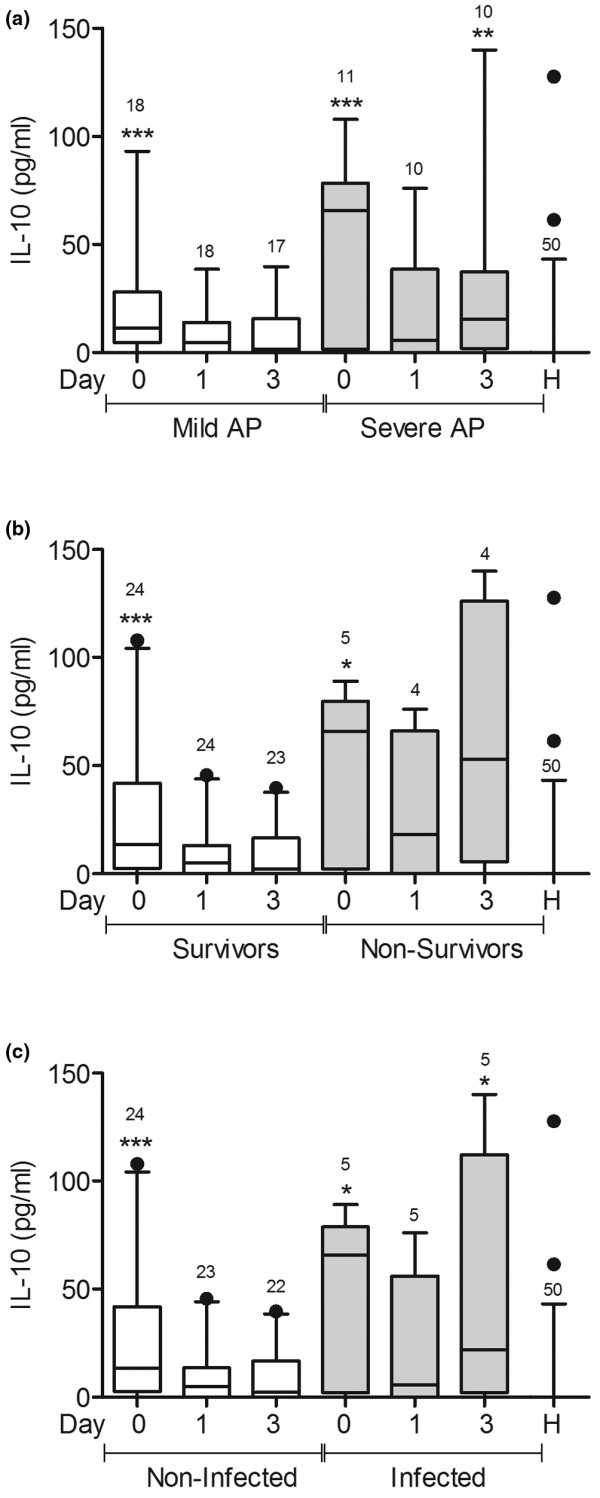
IL-10 concentration was higher in patients with severe AP and in infected patients.  IL-10 concentration was measured in serum from patients with mild acute pancreatitis (AP; n = 18), patients with severe AP (n = 11), and healthy volunteers (n = 19). Samples were taken from patients on admission (day 0) and one and three days later. **(a) **Patients with mild and severe AP, **(b) **survivors and non-survivors, and **(c) **infected and non-infected patients are shown. * *P *< 0.05, ** *P *< 0.01, *** *P *< 0.001; the signs over each bar represent comparisons vs. healthy volunteers (H).

Serum IL-6 and IL-10 concentrations were significantly higher in patients with AP at admission than in healthy volunteers (Figures 3a and 4a). However, the cytokine concentrations declined in patients with mild AP, but remained high in patients with severe AP three days after admission (Figures 3a and 4a). IL-6 concentration was higher in non-survivors than in healthy volunteers three days after admission (Figure 3b), but no difference was observed in IL-10 concentrations at this point (Figure 4b). Both cytokines were increased at admission in uninfected patients but declined three days later (Figures 3c and 4c). In contrast, in infected patients, IL-6 and IL-10 concentrations were higher than in healthy volunteers at admission and three days later (Figures 3c and 4c).

## Discussion

One of the most serious complications of AP is the development of infection. The aim of this study was to measure the levels of TREM-1 and HLA-DR on monocytes and the serum concentrations of IL-6 and IL-10 in patients with AP to determine whether these markers, alone or in combination, can be used in the early identification of patients at high risk of developing severe AP or infection. Our results suggest that TREM-1 expression increases in the presence of inflammation because it was higher in all patients with AP, regardless of the presence of infection. These results support our previous study showing that TREM-1 expression increases after surgery, particularly in patients with preexisting SIRS, but does not correlate with the presence of infection [19]. TREM-1 may be involved in the amplification of the inflammatory response in AP, and its ligand could be an endogenous molecule released during cellular damage associated with AP [24]. Wang and colleagues found higher levels of TREM-1 mRNA in patients with severe AP than in patients with mild AP and healthy volunteers [20]. This seems in contrast to our results, but we measured protein levels and not mRNA, and mRNA levels do not necessarily correlate with protein levels.

TREM-1 can be shed from the surface of monocytes by matrix metalloproteinases [25], and these enzymes are increased in serum in animal models of severe AP [26,27] and in patients with severe AP [28,29]. We found higher TREM-1 expression on monocytes from patients with AP, compared with monocytes from healthy volunteers, but the levels did not differ between patients with mild and severe AP. Perhaps the metalloproteinases found in the serum of patients with severe AP prevented a further increase on TREM-1 expression, but we did not find differences in the concentrations of soluble TREM-1 in serum between patients with mild and severe AP. However, we found that soluble TREM-1 concentration in serum was higher in non-survivors than in survivors. This is in accordance with the study by Yasuda and colleagues, who reported that an increase in the serum concentration of soluble TREM-1, in samples taken within the first 72 hours after the onset of AP, correlated with Ranson score and Acute Physiology and Chronic Health Evaluation (APACHE) II score, and that soluble TREM-1 concentration was higher in patients with early organ dysfunction [30], who have a higher risk of death.

Decreased levels of HLA-DR on blood monocytes and monocyte hyporesponsiveness to PAMPs are suggested as possible causes of the increased predisposition to infection observed in critically ill patients. Satoh and colleagues measured HLA-DR levels on blood monocytes at admission and 7 and 14 days after the onset of AP and found that a persistent decrease in HLA-DR level was associated with the presence of sepsis in later stages of the disease [31]. Mentula and colleagues reported that patients with AP and secondary infections had lower HLA-DR levels on days 14 and 21 of evolution than did healthy volunteers [32]. We measured HLA-DR expression in patients with AP at earlier times and found lower HLA-DR levels in patients with severe AP than in healthy volunteers and patients with mild AP. We also found significantly lower HLA-DR levels in infected patients three days after admission. Our results suggest that the early measurement of HLA-DR level might be useful for identifying patients with AP who are likely to develop a severe form of the disease and who are at high risk of infection.

Ho and colleagues found that HLA-DR expression on less than 52.3% of monocytes on the 10th day after admission correlated with late mortality in patients with severe AP [33]. Our results show that HLA-DR expression at early times does not correlate with patient survival. Mentula and colleagues report that low HLA-DR levels at admission correlate with the development of organ dysfunction in patients with AP [34] but that HLA-DR levels do not differ between survivors and nonsurvivors [32]. Mentula and colleagues also report that organ failure in patients with severe AP could be predicted at admission by a combination of high IL-6 and IL-10 concentrations [32]. In this study, we found persistently high serum concentrations of IL-6 and IL-10 in patients with severe AP and in patients who developed infection, but not in patients with mild AP or uninfected patients.

Our results show that patients with severe AP had low HLA-DR expression on monocytes and high serum IL-10 concentration since the beginning of their disease, which suggest that they presented CARS and could have increased susceptibility to infection. These also suggest that in severe AP, a disease whose early state is a proinflammatory response, an anti-inflammatory response (CARS) develops simultaneously and probably increases the susceptibility to infection.

## Conclusions

Membrane-bound TREM-1 is not useful for differentiating mild and severe forms of AP, or for differentiating infected from non-infected patients with AP, but an increase in TREM-1 expression is associated with the inflammatory process in these patients. Non-survivors had higher soluble TREM-1 concentrations in serum than survivors. In patients with severe AP and in those patients with AP who developed infection, we observed low HLA-DR expression on monocytes and high serum IL-6 concentrations in samples taken at admission and one and three days later. We propose that this pattern could be used to predict the development of severe AP and infection, although further studies are required to confirm this prediction and to determine the appropriate cutoff values. The measurement of HLA-DR expression by flow cytometry is simple and inexpensive, and could be implemented in clinical practice.

## Key messages

• Increased TREM-1 expression on blood monocytes is an indicator of inflammation but not of infection in patients with AP.

• Low HLA-DR expression and high IL-6 concentration could predict severity and infection in samples taken shortly after admission.

## Abbreviations

AP: acute pancreatitis; APACHE: Acute Physiology and Chronic Health Evaluation; CARS: compensatory anti-inflammatory response syndrome; ELISA: enzyme-linked immunosorbent assay; FITC: fluorescein isothiocyanate; HLA: human leukocyte antigen; IL: interleukin; MFI: mean fluorescence intensity; PAMP: pathogen-associated molecular patterns; PE: phycoerythrin; SIRS: systemic inflammatory response syndrome; TNF: tumor necrosis factor; TREM-1: triggering receptor expressed on myeloid cells-1.

## Competing interests

The authors declare that they have no competing interests.

## Authors' contributions

EFO, GRD, and AI conceived of the project. SFF, ADR, GGVG, HRR, and PSF obtained blood samples from patients and followed their clinical evolution. IWB, NEC, and LAP processed the samples. EFO, IWB, RTG, CLM, and AI analyzed the results and wrote the paper.
